# Administration of Bicarbonate Protects Mitochondria, Rescues Retinal Ganglion Cells, and Ameliorates Visual Dysfunction Caused by Oxidative Stress

**DOI:** 10.3390/antiox13060743

**Published:** 2024-06-19

**Authors:** Tonking Bastola, Guy A. Perkins, Viet Anh Nguyen Huu, Saeyeon Ju, Keun-Young Kim, Ziyao Shen, Dorota Skowronska-Krawczyk, Robert N. Weinreb, Won-Kyu Ju

**Affiliations:** 1 Hamilton Glaucoma Center and Shiley Eye Institute, Viterbi Family Department of Ophthalmology, University of California San Diego, La Jolla, CA 92093, USA; tbastola@health.ucsd.edu (T.B.); nhva78@gmail.com (V.A.N.H.); zis003@ucsd.edu (Z.S.); rweinreb@health.ucsd.edu (R.N.W.); 2 National Center for Microscopy and Imaging Research, Department of Neurosciences, University of California San Diego, La Jolla, CA 92093, USAsaeyeonju@gmail.com (S.J.); kkim@health.ucsd.edu (K.-Y.K.); 3 Center for Translational Vision Research, Department of Physiology, Biophysics & Ophthalmology, University of California Irvine, Irvine, CA 92697, USA; dorotask@hs.uci.edu

**Keywords:** oxidative stress, glaucoma, retinal ganglion cells (RGCs), soluble adenylyl cyclase (sAC), mitochondria

## Abstract

Oxidative stress is a key factor causing mitochondrial dysfunction and retinal ganglion cell (RGC) death in glaucomatous neurodegeneration. The cyclic adenosine monophosphate (cAMP)/protein kinase A (PKA) signaling pathway is involved in mitochondrial protection, promoting RGC survival. Soluble adenylyl cyclase (sAC) is a key regulator of the cyclic adenosine monophosphate (cAMP)/protein kinase A (PKA) signaling pathway, which is known to protect mitochondria and promote RGC survival. However, the precise molecular mechanisms connecting the sAC-mediated signaling pathway with mitochondrial protection in RGCs against oxidative stress are not well characterized. Here, we demonstrate that sAC plays a critical role in protecting RGC mitochondria from oxidative stress. Using mouse models of oxidative stress induced by ischemic injury and paraquat administration, we found that administration of bicarbonate, as an activator of sAC, protected RGCs, blocked AMP-activated protein kinase activation, inhibited glial activation, and improved visual function. Moreover, we found that this is the result of preserving mitochondrial dynamics (fusion and fission), promoting mitochondrial bioenergetics and biogenesis, and preventing metabolic stress and apoptotic cell death. Notably, the administration of bicarbonate ameliorated mitochondrial dysfunction in RGCs by enhancing mitochondrial biogenesis, preserving mitochondrial structure, and increasing ATP production in oxidatively stressed RGCs. These findings suggest that activating sAC enhances the mitochondrial structure and function in RGCs to counter oxidative stress, consequently promoting RGC protection. We propose that modulation of the sAC-mediated signaling pathway has therapeutic potential acting on RGC mitochondria for treating glaucoma and other retinal diseases.

## 1. Introduction

Glaucoma is a chronic multifactorial disease with multiple risk factors, including intraocular pressure (IOP) and oxidative stress, and it leads to irreversible blindness on a global scale [1]. Our previous research and other studies strongly suggest that compromised mitochondrial function and metabolic stress caused by glaucomatous insulting factors, such as elevated IOP and oxidative stress, play a crucial role in the degeneration of retinal ganglion cell (RGC) somas and axons in experimental glaucoma [2,3]; this indicates that an impaired mitochondrial network and function are strongly linked to glaucoma pathogenesis. Although the significance of mitochondrial dysfunction and loss in the context of disease is widely acknowledged, the understanding of molecular mechanisms underlying oxidative stress, mitochondrial health, or the prevention of mitochondrial dysfunction in glaucoma remains elusive. 

Cyclic adenosine monophosphate (cAMP) serves as a widely distributed second messenger in the central nervous system (CNS) to facilitate signal transduction [4]. Upon activation, the synthesis and degradation of cAMP are intricately controlled by adenylyl cyclases (ACs) and cyclic nucleotide phosphodiesterases [5]. The activity of cAMP is modulated by diverse effectors, including cAMP-dependent protein kinase A (PKA) [6], guanine-nucleotide exchange proteins [7], and cyclic-nucleotide-gated ion channels [8]. Within the cAMP signaling pathway, ACs are crucial regulators, functioning as enzymes responsible for synthesizing cAMP from adenosine 5′-triphosphate (ATP). At present, there exist ten individual AC genes (AC1–10), encompassing nine mammalian transmembrane ACs (AC1–9) and a soluble AC (sAC; AC10) [9]. 

Current evidence indicates that the sAC/cAMP/PKA signaling pathway is linked to mitochondrial protection, ultimately promoting cell survival in neurons, notably in RGCs [10,11]. The sAC modulates the cAMP activity within the mitochondrial matrix [12,13], and two mitochondrial cAMP pools influenced by sAC are associated with the outer mitochondrial membrane and the intermembrane space of mitochondria [14,15]. Considering that activation of the sAC-mediated cAMP/PKA signaling pathway is crucial for boosting the survival of RGCs and promoting axon growth [11,16], RGC protection from elevated IOP and oxidative stress could serve as a therapeutic approach in addressing glaucomatous neurodegeneration. Importantly, oxidative stress is a critical causative factor in mitochondrial dysfunction and RGC death in glaucoma. 

In this study, we highlight the critical role of sAC in RGC mitochondria protection from oxidative stress. We demonstrate that activating sAC via administration of bicarbonate protects RGCs and ameliorates the degradation of visual function due to oxidative stress by preserving mitochondrial dynamics (fusion and fission), promoting mitochondrial bioenergetics and biogenesis, and preventing metabolic stress and apoptotic cell death in experimental mouse models of oxidative stress. Our findings indicate the therapeutic potential of sAC in preventing impaired mitochondrial dynamics and function, particularly in the context of oxidative-stress-mediated glaucomatous neurodegeneration. 

## 2. Materials and Methods

### 2.1. Animals 

C57BL/6J mice (The Jackson Laboratory, Bar Harbor, ME, USA), classified as adult male and female, were accommodated in enclosed cages, fed with a standard rodent diet, and maintained on a 12 h light/12 h dark cycle. The assignment of mice to either experimental or control groups was performed randomly. Behavioral response and visual function were studied among 4-month-old 15 male and 15 female mice. Research involving animals in ophthalmic vision conducted by the Association for Research in Vision and Ophthalmology follows protocols approved by the Institutional Animal Care and Use Committee at the University of California, San Diego (USA) (IACUC S12063 for mouse and S12067 for rat).

### 2.2. Pharmacological Treatment

NaHCO_3_ was obtained from Sigma-Aldrich (St. Louis, MO, USA). For mouse models of oxidative stress and ischemia–reperfusion, we studied two groups of mice, as follows: a group of C57BL/6J mice treated with regular drinking water (*n* = 15 mice for oxidative stress and *n* = 20 mice for ischemia–reperfusion) and a group of C57BL/6J mice treated with drinking water containing 150 mM NaHCO_3_ (*n* = 15 mice for oxidative stress and *n* = 20 mice for ischemia–reperfusion).

### 2.3. Induction of Retinal Ischemia–Reperfusion by Acute IOP Elevation

C57BL/6J mice were subjected to anesthesia using a mixture of ketamine (100 mg/kg, Ketaset; Fort Dodge Animal Health, Fort Dodge, IA, USA) and xylazine (9 mg/kg, TranquiVed; Vedeco, Inc., St. Joseph, MO, USA) via intraperitoneal (IP) injection. Induction of ischemia–reperfusion by an acute high IOP elevation was performed as previously described [17]. Briefly, a needle (30-gauge) was introduced into the anterior chamber of the right eye, connected via flexible tubing to a saline reservoir. By elevating the reservoir, the IOP was increased to 70–80 mmHg for a duration of 50 min. Contralateral eyes underwent a sham treatment, involving the insertion of a needle into the anterior chamber without saline injection. Following the removal of the cannula, recirculation commenced immediately, and the IOP returned to normal values within 5 min. Subsequently, mice were anesthetized through an IP injection of a ketamine/xylazine cocktail, as previously described, before cervical dislocation was performed at various time points for tissue preparation after reperfusion: 1 day and 4 weeks. The confirmation of retinal ischemia involved the observation of whitening of the iris and the absence of the red reflex in the retina. IOP was measured with a tonometer (icare TONOVET, Vantaa, Finland) during IOP elevation. Non-IOP elevation contralateral control retinas were used as a sham control.

### 2.4. Induction of Retinal Oxidative Stress

To induce oxidative stress, mice received IP injection of PQ (15 mg/kg, Sigma-Aldrich) in saline solution three times during a 1-week period, as previously described [18]. Measurements for the optomotor response and VEP were assessed at 1 week after PQ treatment.

### 2.5. Cell Culture and NaHCO_3_ and PQ Treatment In Vitro

RGCs obtained from 5-day postnatal Sprague Dawley rats were purified through an immunopanning method and cultured in serum-free defined growth medium, following previously established procedures [2]. Approximately 2 × 10^5^, 5 × 10^4^, 1.5 × 10^4^ purified cells were seeded on 60 mm dishes—24-well and 96-well plates—first coated with poly-D-lysine (70 kDa, 10 μg/mL; Sigma-Aldrich), respectively. RGCs were cultured in growth medium containing BDNF (50 μg/mL; Sigma-Aldrich), CNTF (10 μg/mL; Sigma-Aldrich), insulin (5 μg/mL; Sigma-Aldrich), and forskolin (10 μg/mL; Sigma-Aldrich) [2]. 

### 2.6. Western Blot Analyses

Retinas that were collected were homogenized on ice for 1 min using a modified RIPA lysis buffer (Cell Signaling Technology, Danvers, MA, USA), as previously described [2,19]. Proteins (10–20 μg) were run on a NuPAGE Bis-Tris gel (Invitrogen, Waltham, CA, USA) and transferred to polyvinylidene difluoride membranes (GE Healthcare Bio-Science, Piscataway, NJ, USA). To block the membranes, 5% non-fat dry milk and PBS/0.1% Tween-20 (PBS-T) were used for 1 h at room temperature. Subsequently, the membrane was exposed to primary antibodies (Table S1) overnight at 4 °C. Afterward, the membranes underwent PBS-T treatment three times and were then subjected to incubation with horseradish peroxidase-conjugated secondary antibodies (Bio-Rad, Hercules, CA, USA) for 1 h at room temperature. The images were captured using a digital imaging system ImageQuant LAS 4000 (GE Healthcare Bio-Sciences Corp., Piscataway, NJ, USA).

### 2.7. Immunohistochemistry and Immunocytochemistry

Immunohistochemical or immunocytochemical staining was performed on wax sections or cultured cells [2]. Sections from each group (*n* = 4 retinas/group) or cells were used for immunohistochemical analysis. To minimize non-specific background, tissues or cells underwent a 1 h incubation at room temperature in 1% bovine serum albumin (BSA, Sigma-Aldrich) in PBS before being exposed to primary antibodies for 16 h at 4 °C. Following multiple wash steps, the tissues were subjected to a 4 h incubation with the secondary antibodies (Table S1) at 4 °C, followed by PBS washing. Finally, the tissues were counterstained with the nucleic acid stain Hoechst 33342 (1 μg/mL; Invitrogen) in PBS. Images were acquired with Keyence All-in-One Fluorescence microscopy (BZ-X810, Keyence Corp. of America, Itasca, IL, USA). Each target protein fluorescent integrated intensity was measured using ImageJ software. All imaging parameters remained the same and were corrected with background subtraction. 

### 2.8. Whole-Mount Immunohistochemistry and RGC Counting

Whole-mount immunohistochemistry was performed as previously described [2]. Retinas were soaked in PBS with 30% sucrose for 24 h at 4 °C. Following this, the retinas underwent blocking in a blocking solution composed of PBS containing 3% donkey serum, 1% bovine serum albumin, 1% fish gelatin, and 0.1% triton X-100. Subsequently, the retinas were exposed to primary antibodies (Table S1) for 3 days at 4 °C. Following multiple washing steps, the tissues were treated with secondary antibodies (Table S1) for 24 h. Images were acquired with Keyence All-in-One Fluorescence microscopy (BZ-X810, Keyence Corp.). RGCs labeled with Brn3a were counted in two zones, specifically the middle and peripheral regions of the retina (corresponding to three-sixths and five-sixths of the retinal radius) (Table S2). 

### 2.9. Virtual Optomotor Response Analysis

Spatial visual function was assessed using a virtual optomotor system (OptoMotry; CerebralMechanics Inc., Lethbridge, AB, Canada), as previously described [19]. To evaluate visual acuity, tracking was observed when the mouse ceased body movement, and only head-tracking movement was examined. The spatial frequency threshold, indicative of visual acuity, was automatically determined by the OptoMotry HD software (Version 2.1.0) using a step-wise paradigm based on head-tracking movements at 100% contrast. The spatial frequency was initiated at 0.042 cyc/deg and increased progressively until head movement was no longer detected.

### 2.10. VEP Analysis

VEP measurements were conducted following established protocols [19]. The recorded signal was amplified, digitally processed using Veris Instrument software (VERIS™ Science 6.0, Tualatin, OR, USA), exported, and subsequently analyzed for peak-to-peak responses. For each eye, the peak-to-peak response amplitude of the major component P1-N1 in IOP eyes was compared to that of their contralateral non-IOP controls. All the recordings were performed with the same stimulus intensity, and group comparisons were made based on both amplitude and latency.

### 2.11. MitoTracker Red Staining

Cultured RGCs were pretreated with either saline or NaHCO_3_ (25 mM) for 2 h, followed by exposure to PQ (50 μM) for 24 h. Mitochondria in cultured RGCs were labeled by the addition of a red fluorescent mitochondrial dye (100 nM final concentration; MitoTracker Red CMXRos; Invitrogen, Carlsbad, CA, USA) to the cultures and were maintained for 20 min in a CO_2_ incubator. The cultures were subsequently fixed with 4% paraformaldehyde (Sigma-Aldrich) in DPBS for 30 min at 4 °C. Images were acquired with Keyence All-in-One Fluorescence microscopy (BZ-X810, Keyence Corp. of USA). 

### 2.12. MTT Assay

Mitochondrial activity was assessed using 3-[4,5-dimethylthiazol-2yl]-2,5-diphenyl tetrazolium bromide (MTT), in accordance with the manufacturer’s recommendations (Cell Proliferation Kit I; Roche Diagnostics, Basel, Switzerland). In brief, cultured RGCs were pretreated with either saline or NaHCO_3_ (25 mM) for 2 h, followed by exposure to PQ (50 μM) for 24 h. Subsequently, 10 μL MTT stock solution was added to each well, including the negative control. RGCs were treated with 100 μL of the solubilization solution. Following an overnight incubation at 5% CO_2_ at 37 °C, the absorbance (560 and 690 nm) was examined using a microplate reader (SpectraMax; Molecular Devices, San Jose, CA, USA). Each dataset was obtained from multiple replicate wells within each experimental group (*n* = 3).

### 2.13. Transmission Electron Microscopy (TEM) 

For TEM analyses, cultured RGCs were pretreated with either saline or NaHCO_3_ (25 mM) for 2 h, followed by exposure to PQ (50 μM) for 24 h. To initiate fixation, the growth medium was removed, and a primary fixative composed of 2% paraformaldehyde and 2.5% glutaraldehyde in 0.15 M sodium cacodylate (pH 7.4) was added at 37 °C for 2 min. Following TEM tissue preparation as previously described [19], an 80 kV Tecnai Spirit (FEI; Hillsboro, OR, USA) electron microscope was utilized to capture images with a Gatan 2 K × 2 K CCD camera at 2.9 nm/pixel. Quantitative analysis involved measuring mitochondrial profile lengths, and areas were measured with ImageJ (Version 1.54j, National Institute of Health) [19]. The number of mitochondria was normalized to the total area occupied by RGC somas in each image, determined using ImageJ, and the mitochondrial volume density was estimated using stereology [19].

### 2.14. 3D EM Tomography

EM tomography analyses were conducted on a FEI Titan Titan High Base electron microscope operated at 300 kV with nanoscale spatial resolution, and the IMOD package (Version 4.9.10) was used for alignment, reconstruction, and volume segmentation, as previously described [19]. Measurements of both mitochondria and cristae surface areas, as well as volumes, were conducted within the segmented volumes using IMODinfo. Energy calculations were performed using the biophysical model, as previously described [19]. 

### 2.15. OCR Analysis

The OCR was measured using an XFe96 analyzer (Agilent, Santa Clara, CA, USA). Cells were pretreated with either saline or NaHCO_3_ (25 mM) for 2 h, followed by exposure to PQ (50 μM) for 6 h. After measuring the basal respiration, oligomycin (2 μg/mL, Sigma-Aldrich), an inhibitor of ATP synthesis, carbonyl cyanide 4-(trifluoromethoxy) phenylhydrazone (FCCP; 1 μM, Sigma-Aldrich), the uncoupler, and rotenone (2 μM, Sigma-Aldrich), an inhibitor of mitochondrial complex I, were sequentially added to measure maximum respiration, ATP-linked respiration, and spare respiratory capacity. 

### 2.16. Statistical Analysis

For comparison between two groups with a small number of samples related to a fixed control, statistical analysis was conducted utilizing non-parametrical analysis and one-sample *t*-test. For comparison between two independent groups, a two-tailed Student’s *t*-test was performed. For multiple group comparisons, we used either one-way ANOVA or two-way ANOVA, using GraphPad Prism (Version 10, GraphPad, CA, USA). Statistical significance was defined as a *p*-value below 0.05. 

## 3. Results

### 3.1. Administration of Bicarbonate Protects RGCs by Increasing Mitochondrial Biogenesis and Inhibiting BAX Activation in the Retina to Counter Oxidative Stress

**Figure 1:**
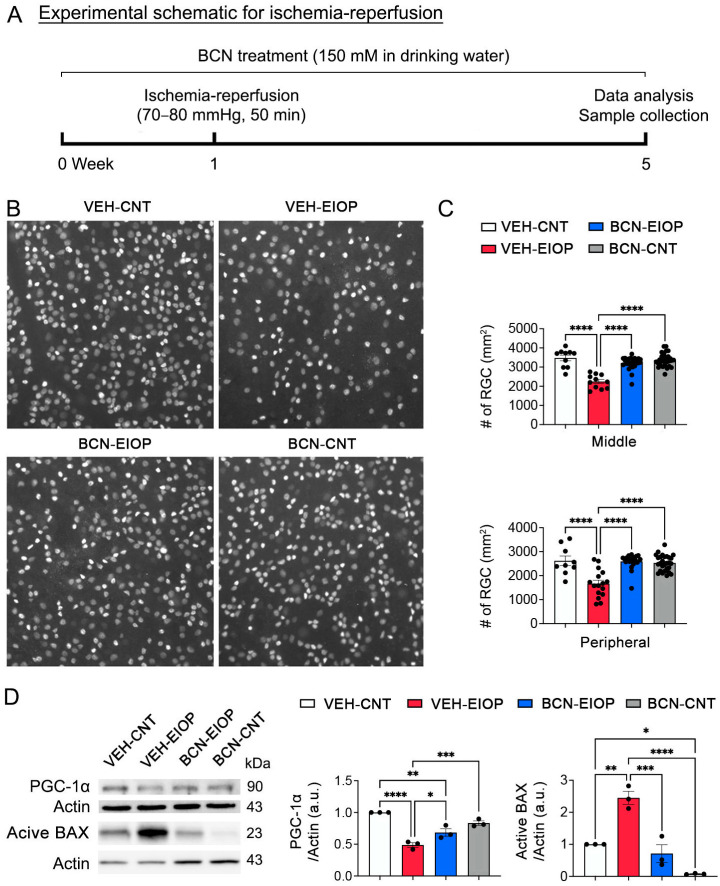
sAC activation protects RGCs by increasing mitochondrial biogenesis and inhibiting BAX activation in the oxidatively stressed retina. (**A**) Diagram for control and bicarbonate (BCN, 150 mM) administration, sample collection, and data analysis. Four-month-old C57BL/6J mice were administered either regular drinking water or drinking water containing BCN for 5 weeks. The drinking water or BCN in drinking water were given weekly. (**B**) Retinal whole-mount immunohistochemistry using BRN3a antibody. Representative images showed BRN3a-positive RGCs in the middle and peripheral areas of the retinas from each group. (**C**) Quantitative analysis of RGC survival. *n* = 5 mice per group. (**D**) PGC1-α and active BAX protein expression in the retina. *n* = 3 mice per group. Error bars represent SEM. Statistical significance was determined using one-way ANOVA test. * *p* < 0.05; ** *p* < 0.01; *** *p* < 0.001; **** *p* < 0.0001. Scale bar: 50 μm. BCN, bicarbonate; CNT, control; EIOP, elevated intraocular pressure. See also Figure S1.

To determine whether sAC activation protects RGCs from retinal oxidative stress, mice were administered regular drinking water or drinking water containing 150 mM sodium bicarbonate (NaHCO_3_) for 1 week before induction of a transient ischemia–reperfusion incidence via acute IOP elevation (Figure 1A). We found that elevated IOP-induced oxidative stress significantly induced RGC loss in the retina compared with non-ischemic control retina (Figure 1B,C). In contrast, we remarkably observed that sAC activation via NaHCO_3_ administration significantly promoted RGC survival in ischemic retinas (Figure 1B,C). sAC activation enhances peroxisome-proliferator-activated receptor-gamma coactivator (PGC1-α), a key regulator of energy metabolism and potential stimulator of mitochondrial biogenesis [20,21]. We found that elevated IOP-induced oxidative stress significantly reduced PGC1-α protein expression in the retina. In contrast, sAC activation remarkably restored the expression level of PGC1-α protein in the ischemic retina (Figure 1D). Moreover, we observed that sAC activation significantly reduced the expression level of active BAX protein in the ischemic retina (Figure 1D). In addition, NaHCO_3_ administration significantly increased cAMP immunoreactivity in the retina (Figure S1).

### 3.2. Administration of Bicarbonate Restores Visual Function That Had Been Reduced by Oxidative Stress

**Figure 2:**
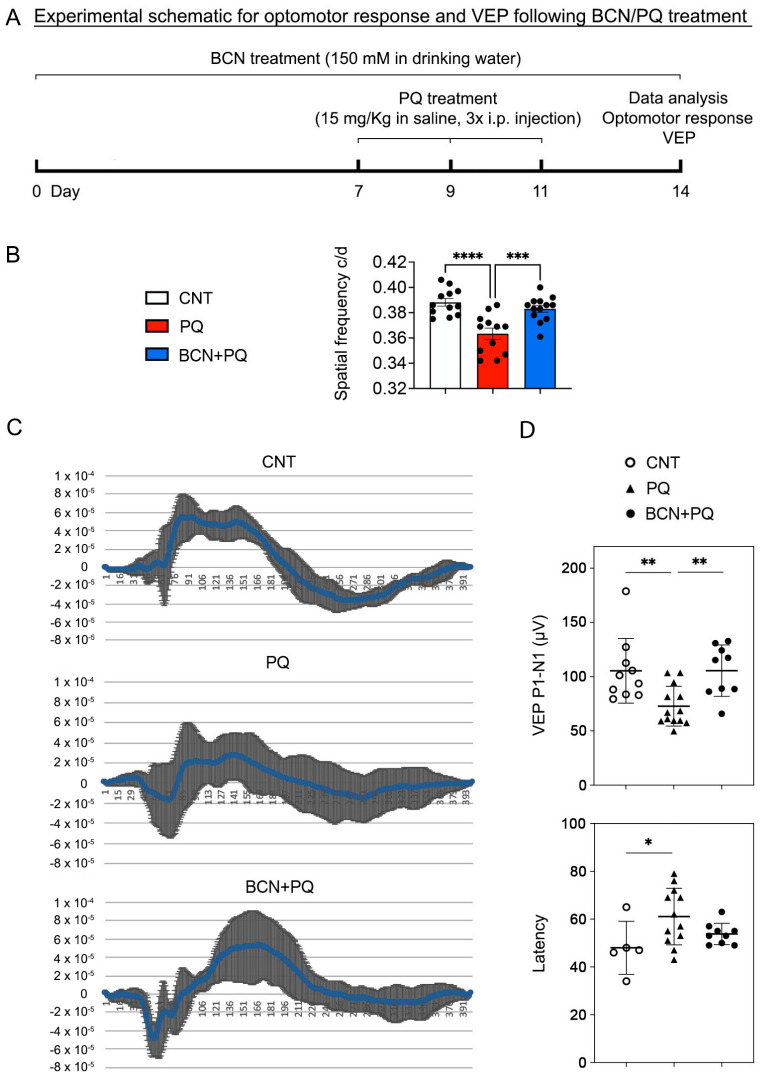
sAC activation restores visual function reduced by oxidative stress. (**A**) Diagram for control and bicarbonate (BCN, 150 mM) administration, sample collection, and data analysis. Four-month-old C57BL/6J mice were administered either regular drinking water or drinking water containing BCN for 2 weeks. The drinking water or BCN in drinking water were given weekly. The mice received PQ (15 mg/kg) via IP injection three times for 1 week after 1 week of BCN pretreatment. (**B**) Visual function test in mice using optomotor response analyses. *n* = 8–9 mice. (**C**) Total recordings of VEP responses among groups. (**D**) Visual function test in mice using VEP analyses. *n* = 9–10 mice. Error bars represent SEM. Statistical significance was determined using one-way ANOVA test. * *p* < 0.05; ** *p* < 0.01; *** *p* < 0.001; **** *p* < 0.0001. BCN, bicarbonate; CNT, control; PQ, paraquat; VEP, visual evoked potential.

To test the effect of sAC activation on visual function recovery from oxidative stress, mice were administered regular drinking water or drinking water containing 150 mM NaHCO_3_ for 1 week before treatment with paraquat (PQ, 15 mg/kg), which is an oxidative stress inducer [22], increasing mitochondrial oxidative damage (Figure 2A). In conditions of oxidative stress, we observed a noteworthy decline in visual acuity, indicated by reduced spatial frequency and visual evoked potential (VEP) P1-N1 potentials, along with increased latency in mice, as measured through the optomotor response and VEP in mice (Figure 2B,C). In contrast, sAC activation remarkably restored the spatial frequency and VEP P1-N1 potentials in mice exposed to oxidative stress (Figure 2B,C). However, there were no statistically significant differences in latency in mice exposed to oxidative stress (Figure 2C). 

### 3.3. Administration of Bicarbonate Inhibits Glial Activation, p38 Phosphorylation, and BAX Activation in the Retina to Counter Oxidative Stress 

**Figure 3:**
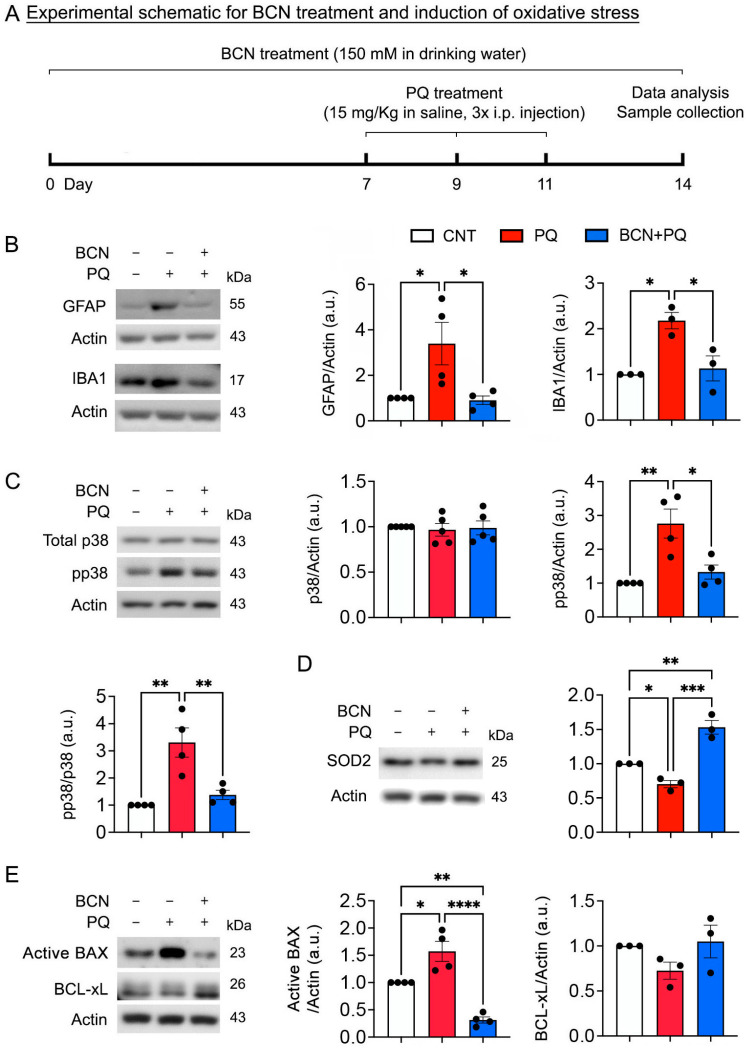
sAC activation inhibits glial activation and MAPK and BAX activation in the oxidatively stressed retina. (**A**) Diagram for control and bicarbonate (BCN, 150 mM) administration, sample collection, and data analysis. (**B**) GFAP and IBA1 protein expression in the retina. *n* = 3 mice per group. (**C**) Total p38 and phospho-p38 (pp38) protein expression in the retina. *n* = 3 mice per group. (**D**) SOD2 protein expression in the retina. *n* = 3 mice per group. (**E**) Active BAX and BCL-xL protein expression in the retina. *n* = 3–4 mice per group. Error bars represent SEM. Statistical significance was determined using one-way ANOVA test. * *p* < 0.05; ** *p* < 0.01; *** *p* < 0.001; **** *p* < 0.0001.

Since oxidative stress induces glial activation (astrocytes and microglial cells) in the retina [23], we determined whether the administration of bicarbonate inhibits glial activation in the retina to oppose oxidative stress (Figure 3A). We observed a significant increase in GFAP and IBA-1 expression in the retina exposed to oxidative stress (Figure 3B). However, sAC activation significantly decreased the expression levels of GFAP and IBA-1 in the retina to oppose oxidative stress (Figure 3B). Oxidative stress is associated with the activation of mitogen-activated protein kinases (MAPKs), including p38, and cell death in the retina [19]. We remarkably found a significant increase in phospho-p38 (pp38) expression in the retina exposed to oxidative stress (Figure 3C). In contrast, the administration of bicarbonate significantly decreased the expression level of pp38 in the retinas exposed to oxidative stress (Figure 3C). Oxidative stress triggers BAX activation and induces apoptotic cell death in the retina [18,24,25]. We observed a significant expression of active BAX but a decreased pattern of BCL-xL in the retina exposed to oxidative stress (Figure 3D). However, the administration of bicarbonate significantly decreased the expression level of active BAX and showed an increased pattern of BCL-xL expression in the retina exposed to oxidative stress (Figure 3D). Superoxide Dismutase 2 (SOD2) is a key enzyme involved in the cellular antioxidant defense system, particularly in scavenging superoxide radicals, which are a hallmark of oxidative stress. We probed for SOD protein expression using Western blot analysis and found a significant decrease in SOD2 expression in the retina exposed to oxidative stress. The administration of bicarbonate significantly restored the expression level of SOD2, indicating a potential reduction in oxidative stress (Figure 3E). 

### 3.4. Administration of Bicarbonate Enhances the Expression of AKAP1 and PKAα, Phosphorylation of DRP1 at Ser637 and GSK3β at Ser9, as Well as Mitochondrial Biogenesis and OXPHOS in the Retina Subjected to Oxidative Stress

**Figure 4:**
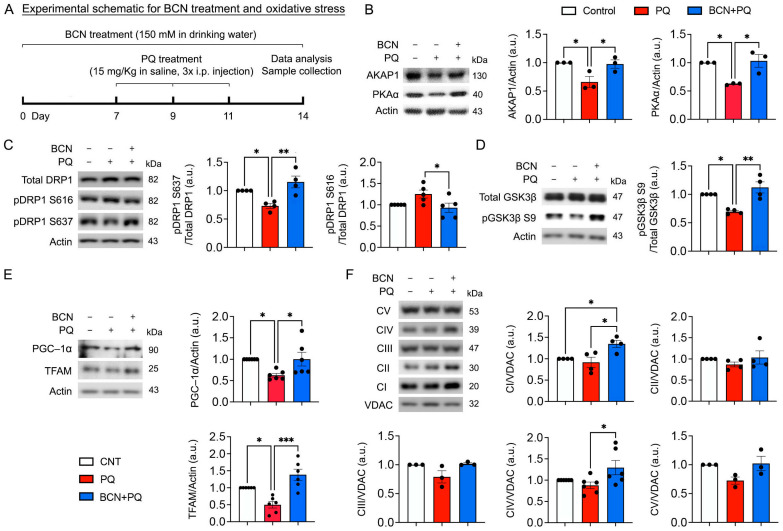
sAC activation enhances phosphorylation of DRP1 and GSK3β, as well as inducing mitochondrial biogenesis and increasing OXPHOS complexes in the oxidatively stressed retina. (**A**) Diagram for control and bicarbonate (BCN, 150 mM) administration, sample collection, and data analysis. (**B**) AKAP1 and PKAα protein expression in the retina. *n* = 3 mice per group. (**C**) Phospho-DRP1 S616 and phospho-DRP1 S637 protein expression in the retina. *n* = 3 mice per group. (**D**) Phospho-GSK3β S9 protein expression in the retina. *n* = 3 mice per group. (**E**) PGC1-α and TFAM protein expression in the retina. *n* = 3 mice per group. (**F**) OXPHOS complex protein expression in the retina. *n* = 3–5 mice per group. Error bars represent SEM. Statistical significance was determined using one-way ANOVA test. * *p* < 0.05; ** *p* < 0.01; *** *p* < 0.001.

The sAC–cAMP–PKA signaling axis contributes to the phosphorylation of mitochondrial proteins, modulation of the oxidative phosphorylation (OXPHOS) system, and regulation of metabolic enzymes [26,27]. We determined whether the administration of bicarbonate via NaHCO_3_ treatment modulates the phosphorylation of mitochondria-related proteins, which are associated with mitochondrial dynamics, in the retina exposed to oxidative stress (Figure 4A). We found that oxidative stress significantly increased total dynamin-related protein 1 (DRP1) protein expression but triggered dephosphorylation of DRP1 at serine 637 (S637) in the retina at 1 week (Figure 4B). However, phosphorylation of DRP1 at serine 616 (S616) was not changed in the retina exposed to oxidative stress (Figure 4B). In parallel, we observed that oxidative stress significantly decreased A-kinase-anchoring protein 1 (AKAP1) and PKAα protein expression in the retina (Figure 4B). In contrast, we found that the administration of bicarbonate significantly restored AKAP1 and PKAα protein expression and phosphorylation of DRP1 S637 and DRP1 S616 in the retina under oxidative stress (Figure 4B,C and Figure S2A). Interestingly, we also found that the administration of bicarbonate did not significantly change the expression levels of the mitochondrial fusion proteins, optic atrophy type 1 (OPA1) and mitofusin (MFN1)/2 (Figure S3). Because DRP1 is also regulated by glycogen synthase kinase 3β (GSK3β) [28,29,30], we further determined whether the administration of bicarbonate modulates phosphorylation of GSK3β at serine 9 (S9) in the retina exposed to oxidative stress. We found that oxidative stress significantly induced dephosphorylation of GSK3β S9 in the retina (Figure 4D and Figure S2B). Based on our previous findings of decreased DRP1 S637 phosphorylation in the retina of AKAP1 knockout (*AKAP1^−/−^*) mice [31], we also found that AKAP1 deficiency significantly induced dephosphorylation of GSK3β S9 in the retina of *AKAP1^−/−^* mice (Figure S4). Impairment of OXPHOS dysfunction is linked to patients with primary open-angle glaucoma [32], and there is supporting evidence indicating that OXPHOS serves as the primary location for the production of mitochondrial superoxide in the presence of PQ [22]. Because the sAC/cAMP pathway is associated with mitochondrial biogenesis and OXPHOS function [10,12,33,34,35], we next examined whether the administration of bicarbonate promotes the expression levels of PGC-1α and mitochondrial transcription factor A (TFAM) proteins and OXPHOS complexes in the retina affected by oxidative stress. We found that oxidative stress significantly decreased PGC-1α and TFAM protein expression in the retina (Figure 4E). In contrast, the administration of bicarbonate increased PGC-1α and TFAM protein expression in the stressed retina (Figure 4E). In addition, we observed that sAC activation enhanced OXPHOS complexes I and IV in the retina against oxidative stress (Figure 4F). 

### 3.5. Administration of Bicarbonate Reduces Mitochondrial Stress by Blocking AMPK Activation in the Retina Subjected to Oxidative Stress

**Figure 5:**
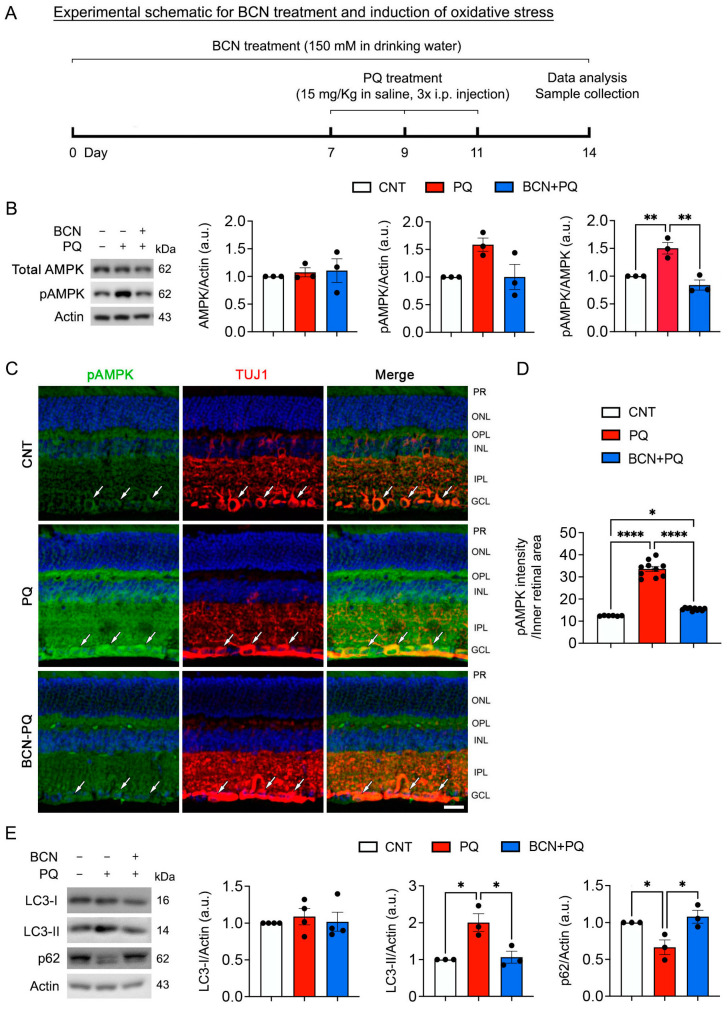
sAC activation promotes mitochondrial function and ameliorates AMPK activation in the oxidatively stressed retina. (**A**) Diagram for control and bicarbonate (BCN, 150 mM) administration, sample collection, and data analysis. (**B**) Total AMPK and phospho-AMPK protein expression in the retina. *n* = 3 mice per group. (**C**) Representative images showed phospho-AMPK (green, arrows) and TUJ1 (red, arrows) immunoreactivities. (**D**) Phospho-AMPK immunoreactive intensity in the inner retina. *n* = 3–5 mice per group. (**E**) LC3 and p62 protein expression in the retina. *n* = 3–4 mice per group. Error bars represent SEM. Statistical significance was determined using one-way ANOVA test. * *p* < 0.05; ** *p* < 0.01; **** *p* < 0.0001. Blue represents Hoechst 33342 staining. Scale bar: 20 μm. GCL, ganglion cell layer; INL, inner nuclear layer; IPL, inner plexiform layer; ONL, outer nuclear layer; OPL, outer plexiform layer; PR, photoreceptor.

5′ AMP-activated protein kinase (AMPK) is a highly conserved energy sensor, and its activation is associated with glaucomatous RGCs [36]. Inactivation of AMPK promotes RGC survival in experimental glaucoma [37]. We determined whether the administration of bicarbonate inhibits AMPK activation in the retina under oxidative stress (Figure 5A). We found a significant increase in pAMPK (Thr172) in the stressed retina (Figure 5A,B). In contrast, we observed that the administration of bicarbonate significantly decreased pAMPK in the stressed retina (Figure 5B). Consistent with an increased pAMPK level, pAMPK immunoreactivity was significantly increased in the RGCs, as well as in the inner retina layers and outer plexiform layer in the stressed retina (Figure 5C,D). However, we remarkably observed that the administration of bicarbonate significantly decreased pAMPK immunoreactivity in the stressed retina (Figure 5C,D). Downregulation of sAC is linked to impaired mitochondrial clearance [38]. However, it is unknown whether sAC contributes to the regulation of mitophagy in the retina exposed to oxidative stress. We further determined whether the administration of bicarbonate reduces mitophagy in the stressed retina. Oxidative stress significantly enhanced microtubule-associated protein 1A/1B-light chain 3-(LC3)-II protein expression but decreased p62 protein expression in the retina. In contrast, the administration of bicarbonate significantly reduced LC3-II protein expression but increased p62 protein expression in the stressed retina (Figure 5E). 

### 3.6. Administration of Bicarbonate Promotes Mitochondrial Biogenesis and ATP Production in RGCs Affected by Oxidative Stress

**Figure 6:**
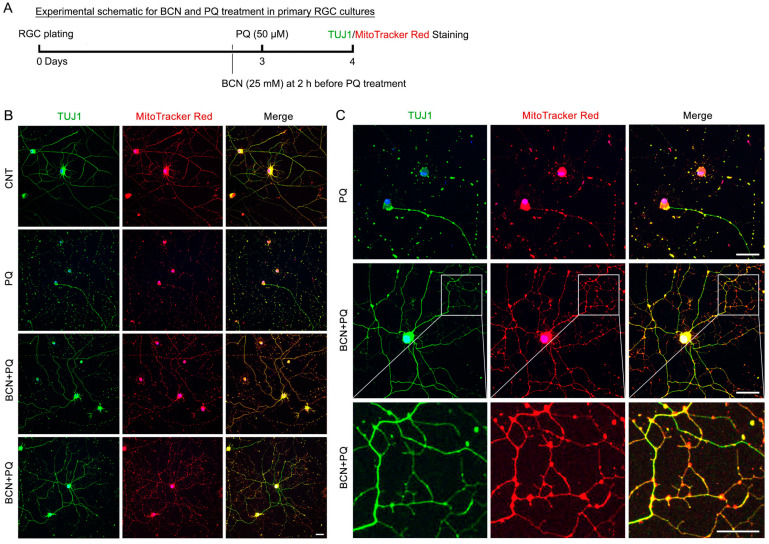
sAC activation promotes neurite outgrowth in oxidatively stressed RGCs. (**A**) Diagram for BCN (25 mM) and PQ (50 μM) treatment, sample collection, and data analysis. Cultured RGCs were pretreated with BCN for 2 h before PQ treatment. PQ treatment lasted 24 h. (**B**,**C**) Representative images showed TUJ1 (green) immunoreactivity, MitoTracker staining (red), and merged image (yellow) in cultured RGCs. Blue represents Hoechst 33342 staining. Scale bar: 10 μm.

sAC/cAMP signaling contributes to neuronal cell survival and neurite outgrowth [11,16]. To determine whether sAC activation protects RGCs and restores mitochondrial function that had been reduced by oxidative stress, we performed MitoTracker Red staining, a marker for mitochondria, and immunocytochemistry using an antibody for β-III-Tubulin (TUJ1), a marker for the RGC soma, axon, and dendrites, at 1 day following PQ treatment (Figure 6A). We remarkably found that MitoTracker Red signals and TUJ1-positive immunoreactivity were diminished in RGCs exposed to oxidative stress (Figure 6B), suggesting that there was a loss of neurites and mitochondria. However, the administration of bicarbonate restored MitoTracker Red signals and TUJ1 immunoreactivity in the stressed RGCs (Figure 6B). More importantly, we observed that the administration of bicarbonate promoted neurite outgrowth in RGCs affected by oxidative stress (Figure 6C).

**Figure 7:**
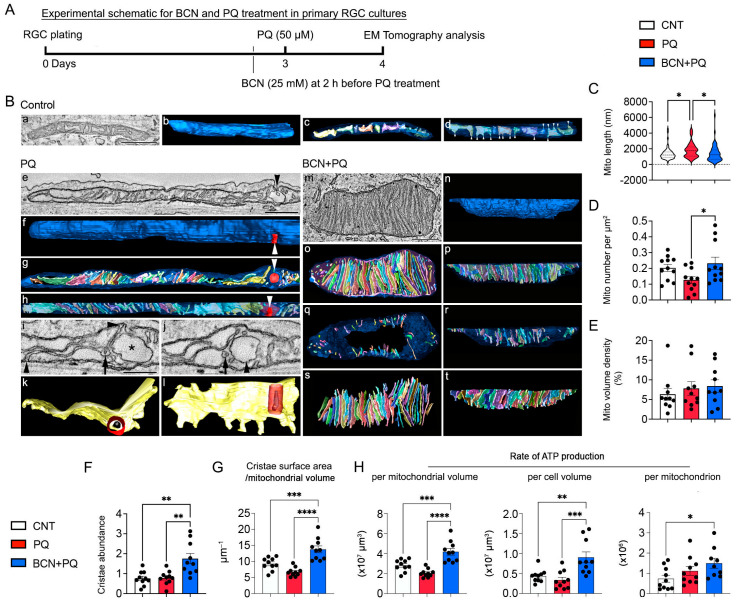
sAC activation promotes mitochondrial bioenergetic function in RGCs to counter oxidative stress. (**A**) Diagram for BCN (25 mM) and PQ (50 μM) treatment, sample collection, and data analysis. BCN was added to cultured RGCs 2 h before PQ treatment. PQ treatment lasted 24 h. (**B**) **Control** (**a**–**d**): Control mitochondria were typically elongated with a slightly condensed matrix (expanded cristae), likely indicating an increased rate of respiration. (**a**) A 1.6 nm thick slice through the middle of a tomographic volume of a representative control mitochondrion. Scale = 500 nm. (**b**) Side view of the 3D-surface-rendered mitochondrial outer membrane shown in blue to demonstrate the tubular nature of control mitochondria. (**c**) Top view of the surface-rendered volume showing the distribution and shape of the cristae (in an assortment of colors), with the mitochondrial outer membrane made translucent to better visualize the cristae. Nine cristae were present in this mitochondrion. (**d**) Side view of the surface-rendered volume showing the mostly lamellar shape of the cristae, but with many extending “fingers” (arrowheads) toward the periphery. **PQ** (**e**–**l**): PQ treatment produced longer mitochondria, yet fewer in number, and abnormal mitochondrial membranes. (**e**) A 2.0 nm thick slice through the middle of a tomographic volume of a PQ-treated mitochondrion. At one place, the mitochondrial outer membrane invaginated inwards to form a chamber (arrowhead). Scale = 500 nm. (**f**) Side view of the surface-rendered mitochondrial outer membrane shown in blue to demonstrate the size and shape of the invagination (arrowhead). (**g**) Top view of the volume showing the distribution and shape of the cristae. This mitochondrion had 35 cristae. Some of the cristae are seen to twist through the volume. The invaginated outer membrane is indicated by the arrowhead. (**h**) Side view of the surface-rendered volume showing the mostly lamellar shape of the cristae with some extending “fingers” toward the periphery. The invaginated outer membrane becomes vesiculated (arrowhead). (**i**) One of the cristae close to the vesiculated outer membrane (*) forms an abnormal tube-within-a-tube structure (arrow). Normal crista junctions are seen at both ends of this crista (arrowheads). Scale = 250 nm. (**j**) The outer membrane forms a completely vesiculated chamber within the mitochondrion (arrowhead), and the tube-within-a-tube structure (arrow) becomes separated from its parent crista, yet remaining close to it as it extends through the volume. (**k**) Top view of the twisted crista emphasized in panels e and f, showing the spatial relation of the tube-within-a-tube structure that extended from the crista (outer membrane: red, inner membrane: charcoal). (**l**) Side view of the same crista showing the extent of the outer membrane (red) and much smaller inner membrane (charcoal) of the abnormal tube-within-a-tube structure. The crista “fingers” extending toward the mitochondrial periphery are clearly seen. **BCN + PQ** (**m**–**r**): Treatment of PQ-exposed cells with BCN restored the number of mitochondria and their length to control levels, yet increasing the crista density. (**m**) A 1.6 nm thick slice through the middle of a tomographic volume of a PQ + BCN mitochondrion that shows the dense packing of cristae. Scale = 500 nm. (**n**) Side view of the surface-rendered mitochondrial outer membrane. (**o**) Top view of the volume showing the distribution and shape of all 109 cristae. The 3D surface rendering further emphasized the high density of cristae. (**p**) Side view of the volume. The cristae were approximately equally divided between lamellar shape and tubular shape. (**q**) Top view of only the 60 tubular cristae, some of which were small. Interestingly, the tubular cristae were arrayed around the mitochondrial periphery and altogether contained only 14% of the total cristae membrane surface area. (**r**) Side view of the tubular cristae. (**s**) Top view of only the 49 lamellar cristae. These are much larger than the tubular cristae and occupy the central portion of the mitochondrial volume. (**t**) Side view of the lamellar cristae. (**C**–**H**) Measurements of structural and functional features of mitochondria. Statistical significance was determined using a one-way ANOVA test. * *p* < 0.05; ** *p* < 0.01; *** *p* < 0.001; **** *p* < 0.0001.

EM tomography showed that mitochondria were typically elongated with a slightly condensed matrix (expanded cristae) in control RGCs (Figure 7A,B(a,d), Video S1). Control mitochondria were observed to be in a slightly condensed state, likely indicating an increased rate of respiration (Figure 7B(a)). The tubular nature of control mitochondria was demonstrated by the 3D surface rendering of the outer mitochondrial membrane (OMM) (Figure 7B(b)). The surface-rendered volume also revealed the distribution and shape of the cristae (Figure 7B(c,d)). The cristae adopted a mostly lamellar shape (Figure 7B(d)). However, oxidative stress produced longer mitochondria, yet fewer in number, and abnormal mitochondrial membranes (Figure 7B(e–i), Video S2). We observed an invagination of the OMM to form an abnormal chamber (Figure 7B(e)). The size and shape of the invagination were demonstrated by 3D surface rendering (Figure 7B(f)). In addition, the surface-rendered volume showed the distribution and shape of the cristae (Figure 7B(g)), being lamellar in shape with some extending “fingers” toward the periphery. An example of a structural defect was seen in an invaginated portion of the OMM becoming vesiculated (Figure 7B(h)). In addition, one of the cristae close to the vesiculated OMM formed an abnormal tube-within-a-tube structure (Figure 7B(i)). Another structural defect was seen in the form of the OMM creating a completely vesiculated chamber within the mitochondrion and the tube-within-a-tube structure becoming separated from its parent crista, yet remaining close to it as it extended through the volume (Figure 7B(j–l)). Remarkably, we found that the administration of bicarbonate increased the crista density (Figure 7B(m–p), Video S3). In addition, the crista shape was roughly equally divided between lamellar (49 cristae) and tubular (60 cristae); yet, the tubular cristae were arrayed around the mitochondrial periphery and altogether contained only 14% of the total cristae membrane surface area (Figure 7B(q,r)). In contrast, the lamellar cristae filled the center of the mitochondrion and were much larger (Figure 7B(s,t)). 

RGCs exposed to oxidative stress experienced significant structural changes. The mitochondrial length was increased (Figure 7C, *n* = 60 mitochondria in 10 RGC somas). The mitochondrial number per cell area was decreased (Figure 7D, *n* = 11 RGC somas). However, oxidative stress did not change mitochondrial volume density, cristae abundance, or crista density (defined as total cristae membrane surface area divided by the mitochondrial volume) (Figure 7E–G, *n* = 10 RGC somas). Interestingly, we found that the administration of bicarbonate significantly reduced mitochondrial length to a level similar to control mitochondria (Figure 7C, *n* = 60 mitochondria in 10 RGC somas) and increased mitochondrial number, again to a level similar to control mitochondria in RGCs subjected to oxidative stress (Figure 7D, *n* = 11 RGC somas). sAC increased cristae abundance and crista density even above control levels (Figure 7F,G, *n* = 10 RGC somas). However, the administration of bicarbonate did not change mitochondrial volume density (Figure 7E, *n* = 10 RGC somas). We next performed energy calculations using a biophysical model based on a 3D structure of the idealized geometrical features of OMM, crista, and a crista junction that derived an approximation of the rate of ATP generation as a linear function of cristae surface area [39]. We observed no significant changes in the modeled rate of ATP production per mitochondrial volume, cell volume, or mitochondrion in stressed RGCs (Figure 7H, *n* = 10 RGC somas). However, we remarkably found that the administration of bicarbonate significantly increased the rate of ATP production per mitochondrial volume, cell volume, and mitochondrion in RGCs (Figure 7H, *n* = 10 RGC somas) subjected to oxidative stress. 

### 3.7. Administration of Bicarbonate Promotes Mitochondrial Respiration in RGCs Subjected to Oxidative Stress

**Figure 8:**
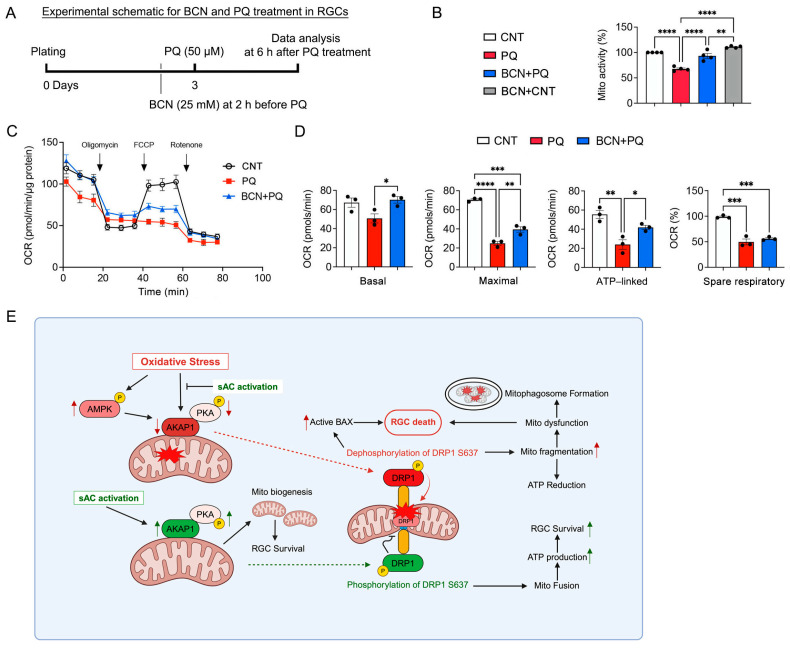
sAC activation promotes mitochondrial bioenergetic function in oxidatively stressed RGCs. (**A**) Diagram for BCN (25 mM) and PQ (50 μM) treatment, sample collection, and data analysis. Cultured RGCs were pretreated with BCN for 2 h before PQ treatment. PQ treatment lasted 6 h. (**B**) The mitochondrial activity was reduced after PQ treatment but protected by pretreatment with BCN. (**C**) OCR changes in cultured RGCs treated with PQ (50 μM) for 6 h. Oligomycin A and FCCP were sequentially added at the indicated time point. Basal respiration indicates the starting basal OCR and the value, which was set to 100%. Maximum respiration represents the ratio between FCCP uncoupled OCR and basal OCR. (**D**) Quantitative analyses of basal, maximum, and ATP-linked respiration, as well as spare respiratory capacity. (**E**) Hypothetical model for protective functions of sAC against oxidative stress and mitochondrial dysfunction in glaucomatous neurodegeneration. The activation of sAC is proposed to mitigate mitochondrial dysfunction, protect RGCs, and alleviate visual impairment caused by oxidative stress induced by ischemia–reperfusion. The activation of sAC is suggested to protect RGCs under oxidative stress conditions by promoting mitochondrial biogenesis and OXPHOS activity while also inhibiting apoptosis. This study establishes a crucial connection between the activation of and the preservation of mitochondrial structure and function in RGCs, offering potential insights into protection against glaucomatous challenges, particularly oxidative stress. *n* = 3 independent experiments. Error bars represent SEM. Statistical significance was determined using one-way ANOVA test. * *p* < 0.05; ** *p* < 0.01; *** *p* < 0.001; **** *p* < 0.0001.

Based on our findings of activating sAC-mediated enhancement of mitochondrial biogenesis and energy production, we examined the effect of sAC activation on mitochondrial activity and respiratory function in RGCs in response to oxidative stress. We measured mitochondrial activity and oxygen consumption rate (OCR) at 6 h following PQ treatment (Figure 8A). We found that the administration of bicarbonate significantly restored mitochondrial activity in RGCs under oxidative stress (Figure 8B). Moreover, we remarkably found that the administration of bicarbonate significantly increased basal and maximum respiration and ATP-linked respiration in RGCs under oxidative stress (Figure 8C,D). 

## 4. Discussion

Oxidative stress plays a pivotal role as a regulator in the neurodegeneration of the retina associated with various ocular diseases, including glaucoma. However, little is known about the potential links and molecular mechanisms between sAC activation and mitochondrial protection in RGCs under oxidative stress. In the present study, we demonstrate the novel protective effects on visual function through activation of soluble adenylyl cyclase (sAC) by administering bicarbonate to RGCs with the goal of countering oxidative stress. The neuroprotective effects of activating sAC were mediated by promoting the expression of AKAP1 and PKAα, enhancing the phosphorylation of DRP1 and GSK3β, mitochondrial biogenesis and OXPHOS activity, as well as by reducing AMPK activation, phosphorylation of p38, and BAX activation. In parallel, activating sAC significantly preserved mitochondrial respiration and structure in RGCs under oxidative stress. We propose that the sAC/cAMP/PKA axis signaling pathway could be a therapeutic target for alleviating mitochondrial dysfunction and RGC death caused by glaucomatous insults, such as oxidative stress.

Increasing the intracellular level of cAMP via electrical-activity-mediated depolarization or bicarbonate exposure promotes RGC survival as well as its axon and neurite outgrowth [11,16]. Of note, the cAMP signaling pathway is linked with the enhancement of PGC1-α activity and the regulation of mitochondrial biogenesis [40,41]. Moreover, intra-mitochondrial cAMP is likely protective against apoptosis [42]. Since excessive mitochondrial fragmentation plays a crucial role in glaucomatous RGC degeneration [2,43], the current evidence unsurprisingly indicates that activating sAC promotes RGC survival and improves visual function by enhancing mitochondrial biogenesis and inhibiting apoptotic cell death. Moreover, inhibiting the activity of DRP1 protects RGCs and/or their axons in glaucomatous DBA/2J and ischemic retina [2,43,44]. Under in vivo oxidative stress conditions, activating sAC restores the expression level of AKAP1 and PKAα protein and enhances the phosphorylation of DRP1 S637 in the retina. Considering that both glaucomatous damage and AKAP1 deficiency result in the dephosphorylation of DRP1 S637 in the retina, leading to excessive mitochondrial fragmentation in RGC somas [2,18], our findings suggest that activating sAC has the potential to protect RGCs by modulating the cAMP/PKA/AKAP1-mediated mitochondrial dynamics in response to oxidative stress.

In the current study, we found that activating sAC restored GSK3β S9 phosphorylation under oxidative stress conditions. GSK3β S9 is a substrate of the cAMP/PKA pathway, and PKA has a role in the phosphorylation and inactivation of GSK3β S9 [45]. Hence, our findings suggest that the sAC-mediated cAMP/PKA pathway has a critical role in protecting retinal cells by promoting the phosphorylation and deactivation of GSK3β S9 caused by oxidative stress. A previous study reported that GSK3β-dependent phosphorylation of DRP1 S693 promoted mitochondrial elongation in response to oxidative stress [28]. Conversely, blocking GSK3β-mediated phosphorylation of DRP1 S40 and S44 prevented neuronal apoptotic cell death triggered by Aβ-induced toxicity [29]. Intriguingly, recent evidence indicated that reducing the GSK3β level promoted RGC survival and increased the number of neurites [46]. Given the role of GSK3β in the cAMP/PKA/DRP1 axis signaling also associated with mitochondrial elongation [30], it is likely that sAC-mediated cAMP/PKA/AKAP1/DRP1 axis signaling is linked to protecting the retinal cells through the regulation of GSK3β S9 phosphorylation and mitochondrial elongation, functioning as a protective mechanism against oxidative stress. However, our findings revealed that activating sAC did not significantly enhance mitochondrial fusion activity in the retina under an oxidative stress condition. Therefore, our findings collectively suggest that activating sAC modulates DRP1 or GSK3β phosphorylation, thereby protecting retinal neurons, including RGCs modified by oxidative stress. 

Accumulating evidence supports the role of cAMP signaling in regulating mitochondrial biogenesis and OXPHOS [10,12,14,26,27,47]. Activation of sAC through bicarbonate or Ca^2+^ increases the cAMP level within the mitochondrial matrix, thereby stimulating OXPHOS activity [48,49]. OXPHOS dysfunction is linked to the pathogenesis of glaucoma [32], and evidence suggests that OXPHOS complex I is the primary site of mitochondrial superoxide production via PQ-induced oxidative stress [22]. In our current study, activating sAC enhanced the activities of OXPHOS complexes I and IV in the retina under oxidative stress. This finding aligns with a prior study, where inhibiting sAC, achieved through the activation of mitochondria-localize type-1 cannabinoid receptors, resulted in decreased mitochondrial cAMP levels, impaired OXPHOS complex I activity, compromised mitochondrial respiration, and lowered cellular ATP content in neuronal cell cultures [50]. Here, our study provides remarkable evidence that oxidative stress induces abnormal mitochondrial fusion activity, leading to damaged cristae and a subsequent reduction in mitochondrial ATP production in RGCs. More importantly, these findings demonstrate that activating sAC preserves RGC mitochondria by promoting mitochondrial biogenesis and ATP production. Notably, these effects provide significant protection to the RGC axon and neurites damaged by oxidative stress. Hence, our findings emphasize a critical role of the sAC/cAMP/PKA signaling axis in mitochondrial protection in RGCs. Activation of cAMP signaling is crucial in mitochondrial biogenesis in mammalian cells [41,51], and a deficiency in sAC has been linked to impaired OXPHOS activity [12]. Hence, our findings suggest that the sAC/cAMP/PKA-mediated modulation of mitochondrial biogenesis could protect RGCs by enhancing the OXPHOS activity in response to oxidative stress. 

As a cellular energy sensor, AMPK is a critical guardian and regulator of metabolic activity that controls normal energy homeostasis [52,53] and has a role in mitochondrial fission under energy stress [54]. sAC is involved in regulating AMPK activity, maintaining mitochondrial function, cellular redox balance, and energy homeostasis [38]. Intriguingly, glaucomatous insults, such as elevated IOP and oxidative stress, are linked to AMPK activation in RGCs of the mouse retina and optic nerve by enhancing AMPK phosphorylation [3,37]. Hence, our findings of activating sAC-mediated inhibition of AMPK activation in stressed RGCs suggest that sAC-regulated AMPK activity contributes to mitochondrial protection in RGCs. Mitophagy or autophagy plays a crucial role in mitochondrial quality control [55], and enhanced mitophagosome formation could be protective in RGCs damaged by glaucomatous insults [43,56]. Indeed, oxidative stress is associated with a reduction in mitophagy via a decrease in p62 in glaucomatous retinal degeneration [57]. Here, activating sAC significantly reversed potential reduction in mitophagy by restoring both LC3-II and p62 levels in RGCs under oxidative stress conditions. Together, these findings strongly imply that activating sAC protects RGCs by alleviating mitochondrial stress caused by oxidative stress.

## 5. Conclusions

In summary, our study establishes an important link between the sAC/cAMP/PKA axis and mitochondrial regulation in RGC protection and vision restoration after glaucomatous insults, such as oxidative stress (Figure 8E). We suggest that the activating sAC-mediated mitochondrial protection holds potential as a therapeutic approach for treating glaucoma. Further studies are required to elucidate the potential mechanism(s) underlying sAC/cAMP/PKA-mediated mitochondrial protection against oxidative-stress-mediated glaucomatous neurodegeneration, including experiments with AKAP1 KO mice. 

## Data Availability

The original contributions presented in the study are included in the article/Supplementary Material, further inquiries can be directed to the corresponding author.

## Supplementary Materials

The following supporting information can be downloaded at: https://www.mdpi.com/article/10.3390/antiox13060743/s1. Figure S1: cAMP protein expression in the retina; Figure S2: Total DRP1 AND GSK3β protein expression; Figure S3: sAC activation did not change the expression level of OPA1 and MFN1 and 2 in the oxidatively stressed retina. Figure S4: sAC activation induces dephosphorylation of in the retina of *AKAP1^−/−^* mice; Table S1: Key Resources; Table S2: Effect of sAC activation on Brn3A-positive RGC survival in the middle and peripheral retina from mice induced by ischemia-reperfusion; Video S1: EM tomography showed that mitochondria were typically elongated with slightly condensed matrix (expanded cristae) in control RGC; Video S2: Oxidative stress produced longer mitochondria, yet fewer in number, and abnormal mitochondrial membranes in PQ-treated RGC; Video S3: sAC activation increased the crista density in PQ-treated RGC.
