# Supplementary material for: Administration of Bicarbonate Protects Mitochondria, Rescues Retinal Ganglion Cells, and Ameliorates Visual Dysfunction Caused by Oxidative Stress
Source: Antioxidants (Basel). 2024 Jun 19;13(6):743. doi: 10.3390/antiox13060743 (PMC11200884; doi:10.3390/antiox13060743)
Supplement: Supplementary file 1 [file antioxidants-13-00743-s001.zip › antioxidants-3033525-supplementary.pdf]

Supplementary Data for

Activating soluble adenylyl cyclase regulates mitochondria, rescues retinal ganglion cells, and ameliorates visual dysfunction caused by oxidative stress

Tonking Bastola[1], Guy A. Perkins[2], Viet Anh Nguyen Huu[1], Saeyeon Ju[2], Keun-Young Kim[2], Ziyao Shen[1], Dorota Skowronska- Krawczyk[3], Robert N. Weinreb[1] and Won-Kyu Ju[1,*]

*Corresponding author: Dr. Won-Kyu Ju, Email: wju@health.ucsd.edu

This PDF file includes:

Supplemental Methods
Supplemental Figures S1 to S4
Supplemental Tables S1 and S2
Supplemental Video Legends S1 to S3

Other supplementary materials for this manuscript include the following:

Supplemental Video S1 to S3

Supplemental Materials and methods

Animals

To investigate the effect of AKAP1 loss, 10-month-old *AKAP1*$^{-/-}$ and age-matched WT mice were used and the *AKAP1*$^{-/-}$ mouse has been described in details before[30]. Research involving animals in ophthalmic vision conducted by the Association for Research in Vision and Ophthalmology follows protocols approved by the Institutional Animal Care and Use Committee at the University of California, San Diego (USA).

Supplemental Figures

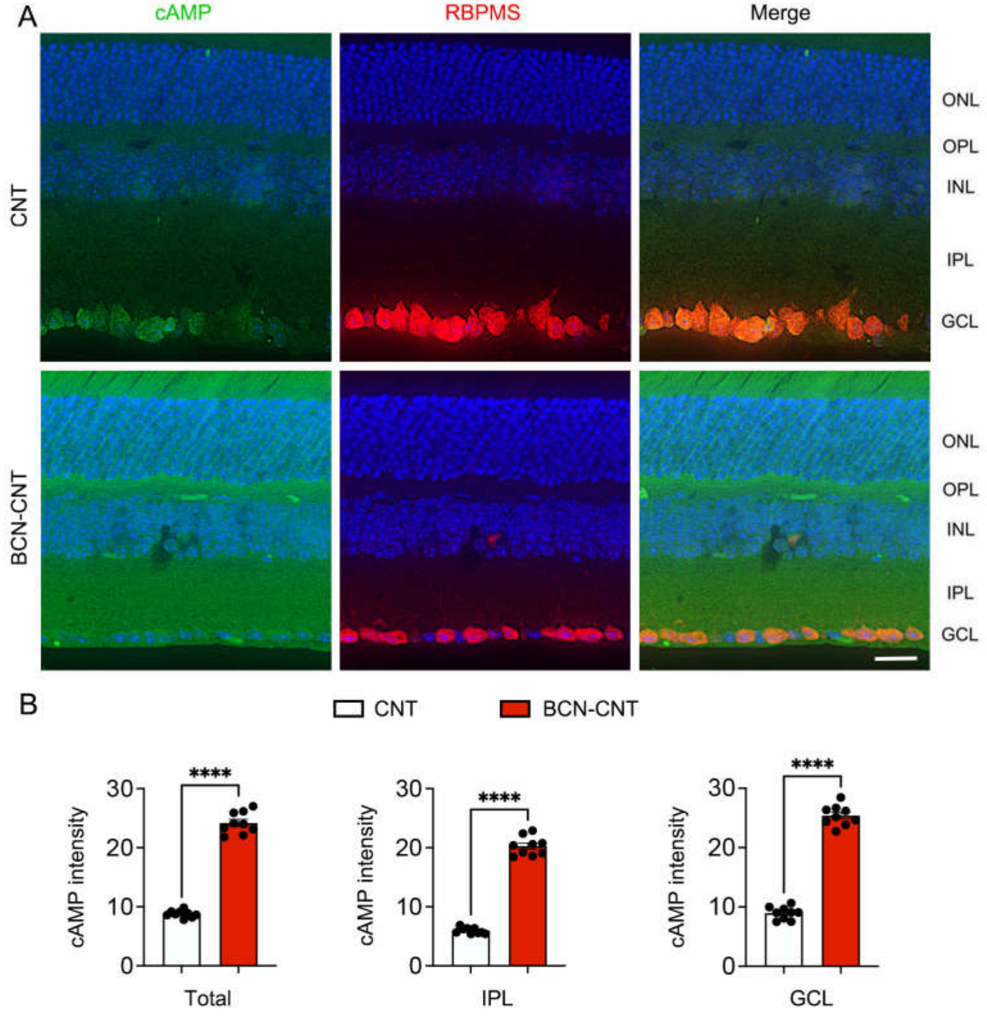

Supplemental Figure S1. cAMP protein expression in the retina. (A) Representative images showed cAMP (green) and RBPMS (red) immunoreactivities. (B) cAMP immunoreactive intensity in the inner retina. *N* = 3 mice per group. Error bars represent SEM. Statistical significance determined using a two-tailed Student's *t*-test. *****P* < 0.0001.

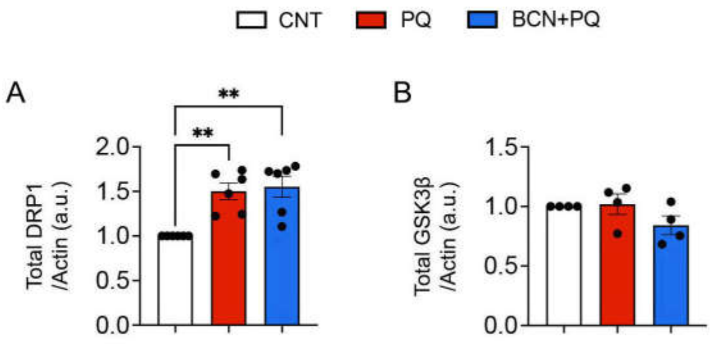

Supplemental Figure S2. Total DRP1 and GSK3β protein expression in the retina. *N* = 4 to 6 mice per group. Error bars represent SEM. Statistical significance determined using one-way ANOVA test. ***P* < 0.01.

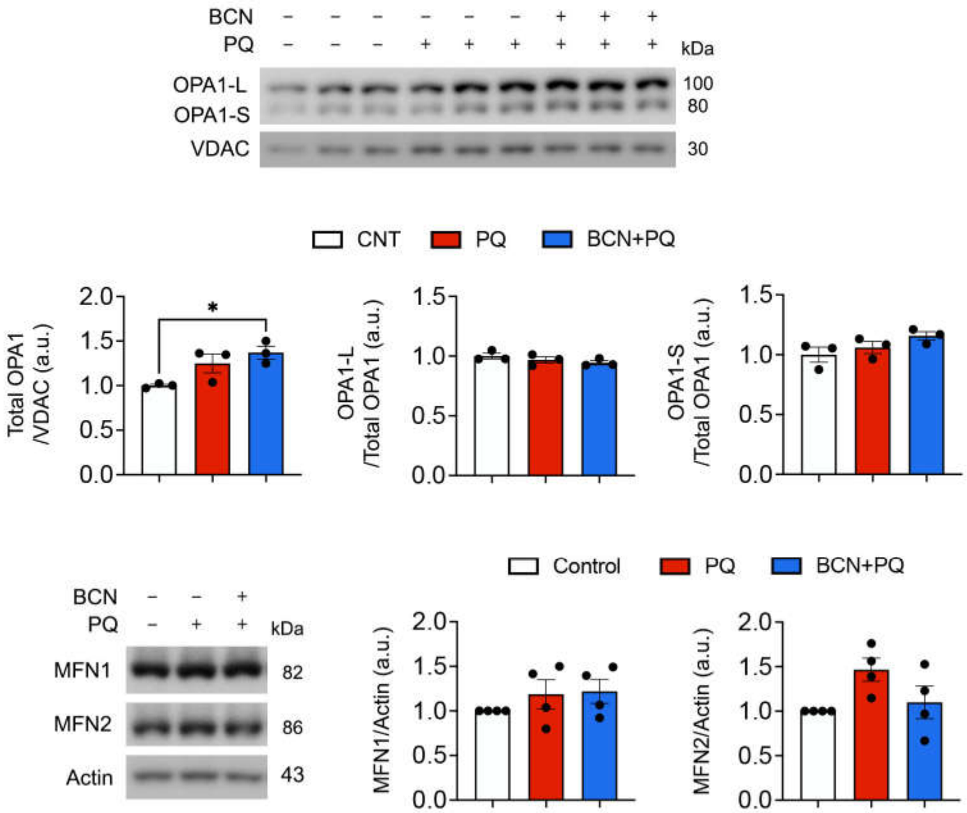

Supplemental Figure S3. sAC activation did not change the expression level of OPA1 and MFN1 and 2 in the oxidatively stressed retina. (A) OPA1 protein expression in the retina. *N* = 3 mice per group. (B) MFN1 and 2 protein expression in the retina. *N* = 4 mice per group. Error bars represent SEM. Statistical significance determined using one-way ANOVA test. **P* < 0.05.

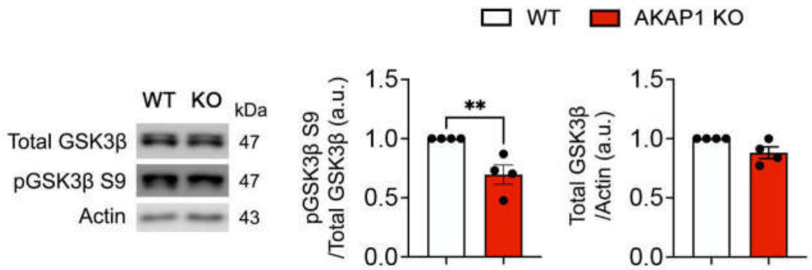

Supplemental Figure S4. sAC activation induces dephosphorylation of GSK3β S9 in the retina of *AKAP1*$^{-/-}$ mice. Total GSK3β S9 and phospho-GSK3β S9 protein expression in the retina. *N* = 4 mice per group. Error bars represent SEM. Statistical significance determined using a two-tailed Student's *t*-test. ***P* < 0.01.

Supplemental Table S1. Key Resources

REAGENT OR RESOURCES	SOURCE	IDENTIFIER
Antibodies	Supplier	Catalogue number
AKAP1	Cell Signaling Technology	CST #5203
AMPK	Cell Signaling Technology	CST #5831
Phospho-AMPK	Cell Signaling Technology	CST #2535
β-ACTIN	Millipore	MAB1501
Active BAX	Santa Cruz Biotechnology	sc-23959
BCL-xL	Santa Cruz Biotechnology	sc-8392
Brn3a	Santa Cruz Biotechnology	Sc-8429
cAMP	Abcam	Ab70280
DRP1	BD Biosciences	#611113
Phospho-DRP1 S616	Cell Signaling Technology	CST #3455
Phospho-DRP1 S637	Cell Signaling Technology	CST #4867
GFAP	Invitrogen	13-0300
GSK3beta	Cell Signaling Technology	CST #12456
Phospho-GSK3b S9	Cell Signaling Technology	CST #5558
IBA1	Wako Chemicals	016-20001
LC3	Cell Signaling Technology	CST #2775
MFN1	Abcam	ab57602
MFN2	Abcam	ab56889
OPA1	BD Biosciences	#612607
OXPHOS	Invitrogen	#458099
p38	Cell Signaling Technology	CST #8690
Phospho-p38	Cell Signaling Technology	CST #4511
p62	MBL International	PM045
PGC-1alpha	Santa Cruz Biotechnology	sc-13067
PKA	Santa Cruz Biotechnology	sc-28315
IBA1	Wako	019-19741
RBPMS	Novus Biologicals	NBP2-20112
TFAM	GeneTex	GTX77852
TUJ1	BioLegend	801202
VDAC	Cell Signaling Technology	CST #4866S
Goat anti-rabbit HRP	Cell Signaling Technology	7074
Goat anti-mouse HRP	Cell Signaling Technology	7076
Alexa Fluor-488 conjugated donkey anti-mouse IgG antibody	Invitrogen	A-21203
Alexa Fluor-568 conjugated donkey anti-mouse IgG antibody	Invitrogen	A-10037
Alexa Fluor-488 conjugated donkey anti-rabbit IgG antibody	Invitrogen	A-21206
Alexa Fluor-568 conjugated donkey anti-rabbit IgG antibody	Invitrogen	A-10042
Chemicals, Reagent and Commercial kits	Supplier	Catalogue number
MTT	Roche	11465007001
MitoTracker Red	Thermo Scientific	M7512
Paraformaldehyde	Sigma-Aldrich	158127
Paraquat	Sigma-Aldrich	36541-100mg
Sodium bicarbonate	Fisher Scientific	144-55-8
Seahorse XF Cell Mito Stress Test Kit	Agilent	103015-100
SuperSignal Chemiluminescent	Thermo Scientific	34580
Experimental models: Strains	Vendor	

C57BL/6J	Jackson laboratory	Stock No: 000664<br>RRID:IMSR_JAX:000664
AKAP1 KO		
Sprague-Dalwley Rat	Envigo (Harlan)	N/A
Software and Algorithms	Suppliers	Site Link
ImageJ	NIH, USA	https://imagej.nih.gov/ij/
Adobe Photoshop	USA	https://photoshop.com
Prism 9	GraphPad Inc.	https://www.graphpad.com/scientific-software/prism/

Supplemental Table S2. Effect of sAC activation on Brn3a-positive RGC survival in the middle and peripheral retina from mice induced by ischemia-reperfusion.

Strain	Treatment	Model	Age (Months)	RGC density per retina ($RGCs/mm^2$)	
				Middle	Peripheral
C57BL/6J	Drinking water	CNT	4	3474 ± 148	2619 ± 169
C57BL/6J	Drinking water	EIOP	4	2243 ± 101	1669 ± 141
C57BL/6J	$NaHCO_3$ in drinking water	EIOP	4	3217 ± 60	2595 ± 67
C57BL/6J	$NaHCO_3$ in drinking water	CNT	4	3362 ± 65	2530 ± 62

All results were reported as means ± SEM. *n* = 5-8 retina wholemounts from 5-8 mice per group. EIOP, elevated intraocular pressure.

Supplemental Video Legend

Supplemental Video S1. EM tomography showed that mitochondria were typically elongated with slightly condensed matrix (expanded cristae) in control RGC.

Supplemental Video S2. Oxidative stress produced longer mitochondria, yet fewer in number, and abnormal mitochondrial membranes in PQ-treated RGC.

Supplemental Video S3. sAC activation increased the crista density in PQ-treated RGC.
